# Intermittent Hypoxia in Childhood: The Harmful Consequences Versus Potential Benefits of Therapeutic Uses

**DOI:** 10.3389/fped.2015.00044

**Published:** 2015-05-19

**Authors:** Tatiana V. Serebrovskaya, Lei Xi

**Affiliations:** ^1^Bogomoletz Institute of Physiology, Kiev, Ukraine; ^2^Department of Internal Medicine, Virginia Commonwealth University, Richmond, VA, USA

**Keywords:** sleep apnea, ventilatory response, bronchial asthma, neurocognitive development, age difference, childhood health, adaptation, hypoxic therapy

## Abstract

Intermittent hypoxia (IH) often occurs in early infancy in both preterm and term infants and especially at 36–44 weeks postmenstrual age. These episodes of IH could result from sleep-disordered breathing or may be temporally unrelated to apnea or bradycardia events. There are numerous reports indicating adverse effects of IH on development, behavior, academic achievement, and cognition in children with sleep apnea syndrome. It remains uncertain about the exact causative relationship between the neurocognitive and behavioral morbidities and IH and/or its associated sleep fragmentation. On the other hand, well-controlled and moderate IH conditioning/training has been used in sick children for treating their various forms of bronchial asthma, allergic dermatoses, autoimmune thyroiditis, cerebral palsy, and obesity. This review article provides an updated and impartial analysis on the currently available evidence in supporting either side of the seemingly contradictory scenarios. We wish to stimulate a comprehensive understanding of such a complex physiological phenomenon as intermittent hypoxia, which may be accompanied by other confounding factors (e.g., hypercapnia, polycythemia), in order to prevent or reduce its harmful consequences, while maximizing its potential utility as an effective therapeutic tool in pediatric patients.

## Introduction

Episodes of intermittent hypoxia (IH) are among typical consequences of immature respiratory control. Particularly, the incidence of IH in the infants with low birth weight would increase progressively over the first 4 weeks of postnatal life and reach a subsequent plateau followed by a slow decline beginning at sixth to eighth weeks. Repetitive cycles of hypoxia/reoxygenation often lead to a pro-inflammatory cascade with resultant multisystem morbidity, including retinopathy of prematurity and impaired growth, as well as cardio-respiratory instability and neurodevelopmental defects (1, 2). Similarly, 15 years ago, Gozal and colleagues had described that IH is the most frequent form of hypoxia occurring in the developing mammal (3), because the maturational process of neural, mechanical, pulmonary, and sleep state-dependent factors would all favor the occurrence of IH during early postnatal life. It has been increasingly recognized that hypoxia, even when short lasting, can modify subsequent respiratory responses to hypoxia and induce a variety of genes whose consequences will persist for much longer periods than the duration of the hypoxic stimulus itself, i.e., functional and adaptive plasticities. The dynamic interactions between the severity, overall duration, and repetitive frequency of IH and the level of maturity of the organs and systems at the time of IH will modify the ventilatory, metabolic, and cardiovascular responses to hypoxia and the adaptive (beneficial) or maladaptive (detrimental) consequences after exposure to IH.

In this review article, we primarily focus on providing an impartial overview on the currently available evidence regarding the functional and structural impact of IH in healthy children or pediatric patients. In addition, the interested readers may find many other important aspects of IH research in adult individuals in two monographs that we recently edited (4, 5).

## Intermittent Hypoxia and Cardiovascular and Metabolic Functions

### Obstructive sleep apnea: Pathological consequences in pediatric patients

One of the most common examples of the negative health impact of IH is obstructive sleep apnea (OSA), which is characterized by brief, recurrent cycles of hypoxia-reoxygenation, typically <60 s in duration. Such repetitive cycles activate sympathetic nervous system and systemic inflammation. When OSA becomes a chronic condition, it often results in adverse physiological effects such as abnormal gas exchange and/or alteration of sleep patterns that impact on health and development (6). In fact, pediatric OSA is not only a very frequent condition affecting 2–4% of all children but also is associated with an increased risk for a variety of end-organ injury and dysfunction (such as accelerated atherosclerosis and endothelial dysfunction) that impose both immediate and potentially long-term morbidities and resultant high healthcare costs (7).

There are a number of reports indicating that alterations in autonomic nervous system function occur in children with OSA, including either an increase in sympathetic nervous system tone and/or responsiveness, or the emergence of sympathetic–parasympathetic imbalance (8–10). A recent prospective cross-sectional study of 26 children with polysomnography-confirmed OSA (versus 30 children in control group) demonstrated that OSA in children was associated with increased lipid peroxidation in an OSA severity-dependent manner, indicated by a positive correlation between plasma oxidized low-density lipoproteins and the apnea/hypopnea index (11).

Interestingly, a recent review by Gozal’s group suggested that IH may not be the primary cause of pathological disorders in pediatric patients with OSA (12). In addition to the component of IH, OSA is also accompanied by hypercapnia, which may be a main contributor to the development of pathological process. The combination of IH, sleep fragmentation, episodic hypercapnia, and increased intrathoracic pressure swings can separately and together activate or amplify the onset and propagation of endothelial dysfunction, atherogenesis, increased systemic inflammation, oxidative stress, and activation of adhesion molecules and coagulation (13, 14). Tam et al. used a piglet model of infant OSA to evaluate circulating IL-6, TNF-alpha, and C-reactive protein following exposure to acute hypercapnic IH (15). The IH protocol consisted of two 90-min sessions of hypercapnic IH with arterial blood sampled before and after each session. The authors concluded that acute hypercapnic IH caused a transient increase in pro-inflammatory cytokine – IL-6 levels, which may have implications for the pro-inflammatory status in pediatric OSA.

Substantial evidence also suggested a major role for IH in altering autonomic nervous system control (12). Both IH and CO_2_ retention may augment sympathetic nerve activity (SNA) via stimulation of both central and peripheral chemoreceptors in adults (16). SNA is higher in infants and young children, and progressively declines through age 5–7 years, followed by a stable period until the beginning of puberty that is associated with increases in SNA (17–19). Until today, little information is available concerning the effect of IH on SNA in children. In addition, severe retinopathy of prematurity was also associated with more variable, longer, and less severe IH events in preterm infants (20). The increased SNA may be driven by two major and potentially interactive pathways as previously proposed, i.e., (1) peripheral chemoreceptor- and baroreceptor-dependent pathway (21–23); and (2) interactions between peripheral and central nervous system (CNS)-located pathways (24, 25). In addition, Zhao et al. suggested that IH differentially regulates plasma membrane Na^+^ channels in the developing brain, depending on duration of IH (26).

### Intermittent hypoxia and cardiovascular and metabolic responses in children

As IH episodes are common among preterm infants, the early postnatal chronic IH exposure may lead to long-term alterations in cardio-respiratory control, such as reduction in baroreflex sensitivity. Reeves et al. have shown that the exposure of rats to chronic IH for the first 30 days of life leads to the substantial structural changes within both nucleus tractus solitarii and ventrolateral medulla (27). A more recent study by Pozo et al. tested the hypothesis that a clustered versus dispersed pattern of repetitive IH during early postnatal life would induce differential long-term alteration in growth and cardiovascular regulation in rat pups from 1 to 7 days of life (28). They found that exposure to both patterns of repetitive IH-induced early growth restriction and exhibited a sustained decrease in heart rate. By contrast, only the clustered paradigm resulted in a significantly lower BP versus controls, while dispersed IH protocol had no effect on blood pressure. Apparently, the repetitive IH during a critical developmental window with either clustered or dispersed IH exposure paradigm contributed to prolonged changes in sympathovagal balance of cardiovascular regulation (28). Pediatric OSA is associated with cardiovascular consequences, including accelerated atherosclerosis and endothelial dysfunction in blood vessels (7).

Severe and chronic IH as occurs during a number of disease states can induce a series of cellular and molecular responses that result in cell injury and death. In response to IH, a number of signaling pathways are involved in oxygen sensing, oxidative stress, metabolism, catecholamine biosynthesis, and immune responsiveness. The cumulative effect of these processes over time can undermine cell integrity and functionality (29). In adult individuals, IH-induced oxidative stress may increase predisposition for metabolic dysfunction by impairing insulin sensitivity and glucose tolerance (30). IH increases serum and liver fatty acid levels due to an increase in sterol regulatory element binding protein-1 (SREBP-1), a transcription factor of lipid synthesis. Endothelin-1 is also an important factor in insulin resistance during IH.

## Intermittent Hypoxia and Cognitive Functions

Hypoxic brain damage is one of the most common perinatal injuries of the CNS. Different degrees of susceptibility of each child to damaging factors and stressors would lead to quite variable outcomes in terms of response to IH and its impact on CNS. Several recent studies indicated perinatal hypoxia as a risk factor for psychiatric disorders like schizophrenia. It is thought that hypoxia prior to or during birth may contribute to alterations leading to the protracted clinical manifestation during young adulthood. A review on various evidences suggested an adverse effect of chronic IH on cognition in childhood, more specifically on development, behavior, and academic achievement (31).

On the other hand, Lima-Ojeda et al. exposed mice during postnatal day 3–7 to two paradigms of chronic intermittent or continuous hypoxia (10% ambient O_2_) (32). They found that neither intermittent nor continuous perinatal hypoxia-induced long-term behavioral alterations. It seems that more severe hypoxic conditions and/or the presence of additional factors (such as genetic risk factors) are necessary for generating long-term behavioral abnormalities. A more recent study by Guo et al. revealed the impact of chronic asthma-induced hypoxia on cognitive function in children using an ovalbumin-induced chronic asthma model in immature mice (33). They reported that chronic asthmatic hypoxia impaired learning and memory ability.

On the other hand, Urschitz and co-workers assessed the association of snoring and IH with poor academic performance in 1,144 of third grade school children (34, 35). A significant relationship between snoring and poor academic performance was found in children without IH, whereas IH did not show an independent association with poor academic performance.

Cai et al. have shown in developing rats that chronic IH exposures across 2 and 4 weeks led to more reference, working, and total memory errors in the 8-Arm radial maze task, and other negative consequences (36). Endoplasmic reticulum stress-related enhancement of neuronal apoptosis was implicated as one of the underlying mechanisms of cognitive dysfunction induced by chronic IH. In pediatric patients, Kirkham and Datta prospectively recorded overnight oxyhemoglobin saturation in 18 children with intractable epilepsy, 6 of whom were currently or recently in minor status epilepticus (i.e., a seizure goes on for 30 min or more) (37). Children with minor status were more likely to have an abnormal sleep study often with desaturation of blood oxygen.

## Effects of Chronic Intermittent Hypoxia on Development in Neonatal Mammals

Despite the fact that chronic IH seems to exert much less impact on the physical growth of animals and humans than chronic constant hypoxia, some evidence suggested that severe IH can also adversely affect the function and development of the organism. In 2008, Farahani et al. reported differential effects of chronic IH and chronic constant hypoxia on postnatal growth and development in mice (38). The postnatal day 2 mice were exposed to constant hypoxia (11% O_2_ in isobaric chamber constantly) or IH (cycles of 4 min 11% O_2_ with 4 min intervals) for 4 weeks. They found that the most severe developmental delay observed under constant hypoxia. Slower weight gain resulted in a 12 and 23% lower body weight in the mice exposed to chronic IH and constant hypoxia, respectively, by postnatal day 30. The decrease in liver, kidney, and brain weight were greater in the constant hypoxia group than IH group. By contrast, the heart weight from chronic constant hypoxia and IH groups was 13 and 33% greater than control (*P* < 0.05), respectively, which was associated with increased size of cardiomyocytes by 12 and 14% (*P* < 0.001) for constant hypoxia and IH mice (38). Similarly, Row et al. (39) exposed rat pups to either room air or IH beginning at postnatal day 10 until day 30 (39). The pups exposed to IH displayed significant spatial learning impairments and increased locomotor activity, which indicated that exposure to IH at postnatal age could induce substantial learning impairment and gender-dependent behavioral hyperactivity in the juvenile rats (39).

Human fetus develops in a profoundly hypoxic environment, which exerts a distant effect on human tolerance to hypoxia that promotes survival advantage under severe hypoxemic stress. The phenomenon and potential mechanisms of neonatal hypoxia tolerance are the subjects for extensive investigations (40, 41). Among the main theories, using a new form of body plethysmograph Cross et al. measured respiratory volume and rate of the newborn infants and first described a positive relationship between body weight and respiratory volume (42). The immaturity of respiratory control in early ontogenesis leads to disruption of metabolic processes in children under hypoxia, which triggers a more ancient way of energy production – anaerobic glycolysis as an adaptive response for surviving in hypoxic conditions (43). In addition, as early as 1921, Benedict and Talbot reported that the daily metabolism adjusted for body surface increases from birth to 1 year of age by 1.6 times. Other researchers have subsequently found that the coincidence in time between the attainment of maximum intensity of respiration and the formation of thermoregulatory mechanism (44). During hypoxia, many newborn mammals, including the human infant, decrease metabolic rate, therefore adopting a strategy common to many living creatures, but usually not adopted by adult humans (45). In 1996, Hochachka and colleagues proposed a biochemical basis for the response of hypoxia-tolerant systems to hypoxia that includes defense and rescue phases (46). The first lines of defense against hypoxia include a balanced suppression of both ATP-demand and ATP-supply pathways, leading to a new steady state even though ATP turnover rates are greatly declined. The ATP demands of ion pumping and protein synthesis are downregulated by channel and translational arrests. In hypoxia-tolerant systems, these arrests activate the gene-based metabolic reprograming “rescue” mechanisms under hypoxia (46).

The development of respiratory lung function in ontogeny is uneven and heterochronic (i.e., a developmental change in the timing of events, leading to changes in size and shape, depending on morphological rearrangement of the lungs and chest and improvement of regulatory mechanisms. The most important stages in the development of respiratory lung function are: neonatal period, up to 1 year, 2–4, 6–7, and 10–11 years. Differentiation of lung tissue is completed by 8–12 years and the growth of tracheobronchial tree ends with the termination of body growth (47). Because of continuous decreasing of specific entropy production, the relative values of lung ventilation and gas exchange (adjusted with body weight or surface) decrease with age (48). One time period of exception is from birth to 1 year, when gradual decrease is not observed, on the contrary, the specific rate of gas exchange and lung ventilation increases (49).

Changes in respiratory reactivity concur with cardiovascular reactivity in young children. In both longitudinal and cross-sectional studies, most investigators found developmental changes with increasing age in heart rate and respiratory sinus arrhythmia – a cardiac index of activation in the parasympathetic branch of the autonomic nervous system (50, 51). Overall, resting HR decreases from infancy to young adulthood and respiratory sinus arrhythmia increases. Autonomic measures at 4 and 8 months of age showed that older infants had more sympathetic activation and less parasympathetic withdrawal in response to stressors than younger infants. On the other hand, a cross-sectional study of older children ages 8–10 and 15–17 years showed no age differences in sympathetic and parasympathetic reactivity (52). There is apparently a slowing of age-related changes in autonomic reactivity as children move developmentally closer to adolescence and adulthood.

Measurement of the ventilatory responses to inhaled hypoxia in children is usually quite difficult due to its methodological and ethical problems. Very few reports are available. It has been known that the carotid chemoreceptors of sino- and cardio-aortic areas are formed in humans at sixth week of fetal life and begin to function before birth. During the first hours of neonatal life, infants are able to increase their ventilation when blood oxygen tension drops. At different stages of ontogenesis, the increase of ventilation in response to hypoxia is provided by unequal changes in breathing modes (53). In contrast to the adult, hypoxic ventilatory response (HVR) in newborns is short and unstable (54). An early investigation from our group in 1977 revealed that inhalation of gas mixture with 14.5% O_2_ for 12 min resulted in an increase in lung ventilation (*V*_E_) during the first minute by 18% in adults and 24% in children of 4–5 years, indicating a stronger initial response in children than those in adults (49). Whereas adults demonstrated a classical biphasic HVR pattern with a subsequent maintain of ventilation at elevated level, the primary HVR in children was short-term and followed by a subsequent reduction of ventilation below baseline and a concomitant decrease in gas exchange, indicating a less pronounced ability to maintain homeostasis of ventilation in young children.

Effects of IH on HVR in developing mammals were well described by Gozal’s group (3). They suggested that despite substantial differences in the acute HVR between adult and immature mammals, IH-induced modifications of HVR are qualitatively similar between immature and adult. Short durations of IH exposures elicit increases in the magnitude of HVR and an attenuated HVR occurs over time if IH exposures are prolonged. However, the neural structures, neurotransmitters, and downstream signaling pathways underlying such biphasic changes in HVR induced by IH are unknown. Using an immature rat model simulating OSA, Moss et al. investigated effects of chronic IH (12% O_2_, 7 h daily) on rats from postnatal day 17 (representing early childhood) through day 33 (representing adolescence) and day 47 (adult) (55). They reported that chronic IH produced long-lasting attenuation in respiratory responsiveness to subsequent acute hypoxia.

It is noteworthy that when assigning hypoxic stress on child and adolescent, it is necessary to first determine their individual HVR for better safety. Such rare cases in HVR were seen in our previous studies. For example, among 20 pairs of monozygotic twins of 10–15 years old whom we tested, there was one male twin who demonstrated complete absence of HVR (56, 57) and the father of the twins had similar defect of sensitivity to hypoxia.

Another early study of our group was conducted on respiration and gas exchange in pediatric patients with chronic pneumonia (58). A significant increase in physiological dead space and reduction of ratio between alveolar and lung ventilation (*V*_A_/*V*_E_) were observed. In the older children (10–14 years), these pathological changes were well compensated by significant hyperventilation that resulted in maintenance of PaO_2_ and gas exchange at the levels of healthy children. However, in the younger children (6–8 years), the compensatory increase in ventilation was not observed, whereas in 4–5 years old patients with protracted pneumonia of stage II, hypoventilation was noted and PaO_2_ was reduced with significantly lower gas exchange rate than age norms. The development of these sick children was also retarded (58). A more recent work showed that HVR during exercise differs between children and adults (59). However, when corrected for body weight, children and adults have similar values for lactic acidosis threshold and maximal oxygen consumption (VO_2_max) during normoxia. Hypoxia significantly lowered lactic acidosis threshold and VO_2_max in both children and adults. Metabolic efficiency was similar among the two age groups and unaffected by hypoxia.

Intermittent hypoxia during development has also been implicated as a potent inducer of respiratory plasticity (60). The altered ventilatory pattern induced by IH is distinct from other stimuli and elicits markedly different responses in the developing mammal as compared to the adult. Exposures to either hypoxia or hyperoxia during early postnatal life may lead to significant modifications of neural function during adulthood. For example, suppression of peripheral arterial chemoreceptor activity via exposure to hyperoxia (60% inspired oxygen) during the first month of rat life led to significant reduction in the number of unmyelinated axons in the carotid sinus nerve and petrosal ganglion in the adult rat that are accompanied by substantial attenuation of the HVR at 3–5 months of age but not at 15 months. By contrast, when adult rats are exposed to hyperoxia, no changes in HVR characteristics occur, indicating that the persistent plasticity changes in the pathways underlying HVR are unique to the interaction between environmental stimulus and a critical developmental window (61).

Furthermore, Paton et al. reported in 1989 that both hypercapnic and hypoxic responses during wakefulness were defective in pediatric patients, 6–11 years of age, with congenital central hypoventilation syndrome (CCHS). These sick children had no subjective sensation of dyspnea or discomfort, but no significant change was found from the baseline levels of ventilation in response to either stimulus. The researchers speculated a defect caused by CCHS in central integration of the central and peripheral chemoreceptor signals (62). Thus, long-lasting plasticity of neural networks underlying respiratory control is more likely to occur during early and more plastic stages of development. Application of IH during these critical stages of development likely regulates adaptive processes that could be used to our advantage for preventive or therapeutic purposes as we further elaborate in the following sections.

## Therapeutic Uses of Intermittent Hypoxia in Pediatric Practice

Hypoxic training or conditioning can be traced back from the traditional medical remedy used in ancient time. For example, in the Carpathian mountain region, children who suffered from asthmatic bronchitis were ranged on foot, during 7 days successively, on a high mountain with ingestion of high-altitude herbal tea. The children had recovered. Similarly, a common yogic treatment of various diseases in India, so called “nisshesha rechaka pranayama,” involves breath holding at residual volume, which produces brief IH that triggers the adaptive mechanisms (63). Nevertheless, the scientific basis for the observed beneficial effects of the so-called intermittent hypoxic training/therapy (IHT) on human organism remains elusive despite the extensive investigations over the past five decades (4, 5). In the following sections, our discussions focus mainly on the abundant experience and evidence of IHT implementation in pediatric practice, which have been almost exclusively reported by Ukrainian and Russian physicians and researchers since 1970s (58, 64–67). Based on these studies, various highly specialized IHT equipment and portable devices such as “Hypoxicator” (68) have been invented and developed, including those for pediatric patients, although there are still a number of remaining issues related to the complexity and non-standardization of various IHT protocols reported in different studies. In addition, further thorough risk-versus-benefit evaluations of IHT will address some fundamental ethical concerns on the IHT practice in pediatric patients.

### Bronchial asthma

Most abundant information for the therapeutic use of IHT has been found in the treatment of bronchial asthma (BA). For example, a study by Anokhin et al. (64) applied IHT with a normobaric hypoxic stimulation with four sessions of 5 min 12-15% O_2_, followed by 5 min normoxic interval, for 10 days in 200 children aged 4–14 years who suffered from asthma (64). Positive effects were seen in 85% of subjects in the IHT group and only in 25% of the sham control group. In children with mild BA, a complete discontinuance of asthma attacks was observed and a significant improvement was also observed in patients with moderate to severe forms of BA without medication (64). The beneficial effects lasted for an average of 4 months after IHT. By contrast, in patients with the severe form of BA, only small or no improvement was found. In the hormone-dependent form of BA, efficacy of IHT was also unsatisfactory (69).

Such therapeutic effects of IHT were subsequently confirmed by several other research groups in Ukraine and Russia (64, 67, 70, 71). Among these works, in 1990, Meerson and co-workers studied the effects of adaptation to IH on immune status and neuro-humoral regulation in pediatric and adult patients with BA, allergic dermatoses, and autoimmune thyroiditis (66). They reported that the IH adaptation facilitated normalization of humoral values of immunity in allergic and autoimmune disorders and led to increased serum levels of immunoglobulins, while the level of circulating immune complexes reduced. These beneficial changes of the immune system were associated with an increase of the reserve potency of the hypothalamo-hypophyseal-adrenal and sympathoadrenal systems as well as a reduction in blood histamine levels.

In recent years, there was a revitalized interest in the use of IHT in children with BA. For example, researchers from Brazil studied 48 adolescents (12–14 years of age) under three conditions: mild intermittent asthma; mild persistent asthma; and control (72). They concluded that adolescents with mild persistent asthma have a greater capacity to adapt to hypoxia than do those with other types of asthma. In addition, Serebrovskaya et al. used IHT for treatment of children (aged 9–13 years) with persistent atopic BA in moderate form without the signs of respiratory insufficiency. The subjects in experimental group underwent IHT alone with regular drug treatment and the control group received the same medical treatment, but not IHT (73). Before and next day after the 2-week session of IHT, individual cardio-respiratory reactions to hypoxia were investigated where normobaric hypoxia was administered with a portable device “Hypoxotron–Complex,” a modified closed spirometer with CO_2_ absorption (68). The initial inspired gas was atmospheric O_2_ (20.9%) and inspired O_2_ fell to 12% after 60–90 s of rebreathing, and then O_2_ was added gradually to the device to maintain inspired O_2_ at 12% for the remaining 3.5–4 min with a final arterial O_2_ saturation (SaO_2_) typically 89–92%. All children easily tolerated the hypoxia periods without any untoward effects. Each IHT session consisted of four 5–7 min hypoxic periods, followed by 5 min interval with room air inspiration. A significant decline in breath shortness and feelings of chest congestion were noted in the patients of IHT group, with other symptoms such as cough and attacks of asphyxia diminished or disappeared. No significant changes in airway conductance before and after IHT. By contrast, significant differences in hypoxic ventilatory sensitivity were found as a result of IHT, suggesting that adaptation to IH caused considerable augmentation in ventilatory response to hypoxia, likely due to both central and peripheral mechanisms as previously proposed (74, 75). Meanwhile, the heart rate response to hypoxia became less pronounced and SaO_2_ fell less at 12% O_2_, indicating IHT improved efficiency of cardiovascular system in supporting oxygen supply during hypoxia (73).

Since the development of inflammatory process in lungs is usually associated with activation of free radical oxidation and decline of antioxidant enzymes activity (76), the role of antioxidant enzymes in adaptation to IHT has been a key subject for investigation in pediatric BA patients. Nesvitalova et al. examined the effect of a 10-day IHT on the mRNA expression and protein content of antioxidant enzymes such as Cu,Zn-superoxide dismutase (Cu,Zn-SOD), catalase (CAT), and glutathione-S-transferase (GST) in blood leukocytes of asthmatic children (77). They reported that following IHT, Cu,Zn-SOD protein content in leukocytes did not change significantly, but Cu,Zn-SOD mRNA expression increased by 33%. Conversely, GST protein synthesis increased by 90%, but its mRNA expression was invariable. Both protein content and mRNA of CAT increased by 37 and 13%, respectively. Furthermore, IHT altered mitochondrial enzymes such as succinate dehydrogenase (SDG) and alpha-glycerophosphate dehydrogenase (GPDG) in asthmatic children. In 2002, Kurhaliuk et al. demonstrated a strong correlation between the individual hypoxic sensitivity and mitochondrial enzymes activities of GPDG in rats (78). In 2012, Serebrovskaya et al. have shown that in children with decreased HVR, elevated basal SDG and GPDG activities were observed and greater increase was found after IHT (73). Similarly, they measured SDG and GPDG activities in peripheral lymphocytes of asthmatic children (9–13 years old). Both SDG and GPDG activities significantly increased under IHT by 78 and 42%, respectively.

### Brain function

There are few clinical observations made by Ukrainian and Russian researchers regarding the effects of IHT on CNS in children. Among the recent studies, Yatsenko et al. investigated the effects of IHT on CNS function and cerebral circulation in pediatric patients with cerebral palsy (79). The 87 sick children (ranged from 9 months to 12 years of age) were examined before and immediately after the IHT course via inhalation of normobaric hypoxic gas mixture (12% O_2_). Each cycle included 15 min of hypoxia alternated by 5 min of normoxia. The number of IH cycles gradually increased from one to three per day and the entire course of IHT lasted 10 days on average. The authors observed stable positive effects of IHT on the motor status in 94% of the patients and positive dynamics of spectral EEG components were seen in 70% of the patients. Doppler-detected brain hemodynamics was also normalized in 85% of the children who underwent IHT (79).

Borukaeva recently reported a study on brain bioelectrical activity, mental fitness, coordination of movements among 250 healthy young individuals (8–21 years of age) (80). The subjects’ EEG was recorded while breathing air or hypoxic gas mixture via “Bio Nova 204” hypoxicators made in Russia. They found that the changes in bioelectric activity of the brain under short-term hypoxia (manifested with the increased index and amplitude of alpha, theta, and delta waves of EEG) were similar among the groups of children (8–12 years), adolescents (13–16 years), and young adults (17–21 years). In the children group, the changes in alpha and theta waves were more sensitive indicators of hypoxia than other EEG waves. These data suggested an increased cortical impact on the background of increasing limbic system activity. On the other hand, the adolescents showed a greater reduction in mental performance and motor coordination during hypoxic exposure as compared with the other age groups. They showed an increased time for the maze passage, the number of touches and goes beyond the maze, decreased concentration and irradiation of excitatory and inhibitory processes, breach of their forces and mobility (80). These results suggest that under hypoxia the children or young adults usually have increased cortical activity, whereas the adolescents have enhanced activity of subcortical structures. The age dependent-influence on CNS should be taken into account when designing IHT protocols with the most optimal condition, in order to prevent any undesirable complications of IHT.

In addition, a recent study demonstrated that IHT (5-min episodes of 10.5% O_2_ with 5 min normoxic intervals, three exposures per week for 10 weeks) upregulates pro-plasticity molecules without evidence for CNS pathology in adult rats (81). The authors suggested that IHT can be a useful therapeutic tool in treating disorders that cause respiratory insufficiency, such as spinal injury or motor neuron disease (81). However, it remains to be determined whether such beneficial effects of IHT can be reproduced in immature bodies.

### Metabolic effects

The therapeutic use of IHT in treatment of metabolic disorders such as obesity had not been investigated until recent years. For instance, in 2014, Wang et al. designed and initiated a new randomized controlled trial to assess the effectiveness of a 4-week IH exposure plus conventional exercise training and diet intervention for inducing short- and long-term weight loss in obese adolescents (Clinical trial registration No. ChiCTR-TRC-14004106) (82). They planned to allocate 40 obese boys and girls (11–15 years old) into control group (sleep in normal conditions) and hypoxia group (sleep in a normobaric hypoxia chamber, simulating the “sleep high and train low” IHT mode). Results obtained from this study would potentially provide important evidence for the potential use of IHT in a weight loss intervention program among obese children and adolescents. Clarification of the mechanisms leading to weight loss in “sleep high and train low” protocol such as appetite regulatory effects could provide new information for the development of new strategies in combating obesity.

## Potential Confounding Factors for Intermittent Hypoxic Training in Children

### Hypoxia-induced polycythemia

An increased production of red blood cells (i.e., polycythemia) is one of the common adaptive responses of the body to hypoxia for improving oxygen transport capacity from the lungs to the target organs/tissues. Hypoxia stimulates the secretion of erythropoietin in kidneys and this hormone in turn stimulates the production of erythrocytes in bone marrow. It was suggested that erythropoiesis is an essential mechanism for long-term acclimatization to hypoxic condition (83). In fact, polycythemia was observed in humans following chronic intermittent exposures to hypoxic environment at high altitude (84–86). A recent study reported that intermittent mining activity in the Andes at 4000 m altitude (i.e., from 4th to 20th day of each month worked at high-altitude mines followed by a resting period of 14 days at sea level) stimulates the production of red blood cells in bone marrow (87). These results in adult humans are in contrast to an earlier study of Quechua Indians children, living at 4200 m altitude (88), which provided no support for the long-held belief that altitude hypoxia provokes a dramatic compensatory polycythemia in healthy adults.

In addition, although polycythemias or erythrocytoses in childhood and adolescence at sea level are very rare, the neonatal infants who are small for gestational age or affected by maternal gestational diabetes are at high risk for developing polycythemia (89). Primary polycythemias are characterized by acquired somatic or inherited germ-line mutations expressed within hematopoietic progenitors that cause increased accumulation of red blood cells. Secondary erythrocytoses are driven by hormonal factors (predominantly by erythropoietin) extrinsic to the erythroid compartment (90). With placental insufficiency, there may be chronic or acute fetal hypoxia resulted from birth asphyxia and hypothermia, neonatal hypoglycemia, polycythemia, and coagulopathy (91). Lee et al. described a family case of inherited CCHS, who was accompanied by hypoxia and hypercapnia and polycythemia with a hematocrit level of 70% (92). Furthermore, IH resulting from sleep apnea was suspected to induce polycythemia. However, Solmaz et al. recently suggested that OSA rarely causes secondary polycythemia (93). Similarly, King et al. commented that OSA does not lead to clinically significant erythrocytosis (94). There is also no published evidence suggesting development of polycythemia in the children undergoing IHT for therapeutic purposes, which involves intermittent brief inhalation of hypoxic gas mixtures. Nevertheless, the issue of polycythemia should be considered as a potential confounding factor to be monitored in future practice of IHT in pediatric patients.

### Hypoxia-triggered disturbance in carbon dioxide homeostasis

Carbon dioxide (CO_2_) is an important gaseous molecule that maintains whole body homeostasis as well as cellular signaling. CO_2_ accumulates in the tissues during each episode of airway obstruction in sleep apnea or breath holding leading to acidemia. To the contrary, during IHT (e.g., high altitudes, hypobaric chamber, or inhalation of hypoxic gas mixtures), systemic hypoxia elevates pulmonary ventilation leading to hypocapnea and alkalemia. The physiological consequences of hypocapnea versus hypercapnia during IHT remain partially understood. At cellular levels, such changes in arterial CO_2_ levels may affect vascular dynamics via activation or inactivation of vasoactive factors such as nitric oxide, angiotensin II, endothelin, and bradykinin (95).

On the other hand, Zhang et al. recently investigated experimental hypocapnia and hypercapnia following 14-day sessions of IHT with 10% inspired O_2_ and they reported that the repetitive normobaric IH exposures significantly diminished variations of cerebral perfusion in response to both hypercapnia and hypocapnia without compromising cerebral tissue oxygenation in adult humans (96). Similar results were also communicated recently by Fan and Kayser (97). Another study by Snow et al. in rats indicated that hypocapnic but not eucapnic IH increases hematocrit and causes a more profound increase in right ventricular mass than those who underwent eucapnic IH (98). Taken together, there are very limited data on the role of hypocapnia or hypercapnia in the adaptive process to IH or IHT in adults and virtually no report in pediatric individuals. Therefore, at the present time, we cannot give concrete advice on how to manipulate the CO_2_ levels during IHT for avoiding adverse effects and/or enhancing therapeutic efficacy of IHT. There is little doubt that the concomitant changes in arterial CO_2_ levels during IH exposures can have profound impact on the neurologic and other cellular outcomes (detrimental or beneficial), considering the critical regulatory role of CO_2_ homeostasis in the body systems. This important question should be carefully addressed in future investigations.

## Concluding Remarks

Taken together, the above-discussed studies in healthy children and pediatric patients have indicated that IH can profoundly trigger either adaptive or maladaptive responses in childhood, which has impact on the structural and functional development in multiple body organs and systems. Whereas more severe regimens of IH induce pathological outcomes, the well-controlled and proper regimens of IHT can produce therapeutic effects against various chronic diseases in children, including BA, allergic dermatoses, autoimmune thyroiditis, cerebral palsy, and obesity. Many interesting emerging fields of IH research such as stem cell biology should be further explored. For example, Bhaskara et al. recently reported that IH enhanced stem-like characteristics and suppressed differentiation propensities in neuroblastoma cells (84). Serebrovskaya et al. have shown that IH mobilizes hematopoietic progenitors and augments cellular and humoral elements of innate immunity in adult men (85).

It is noteworthy that the different stages of child development and various degrees of maturity have great impact on the individual response to IH. Such an age-dependence is not fully characterized in this review due to the lack of published evidence. The short- and long-term effects of IH on the modulation of neurotransmitter release, receptor binding and expression, intracellular signaling cascades, transcriptional regulation, and gene expression as a function of animal maturity are almost completely unknown. As Waters and Gozal pointed out that the responses to IH vary according to the point within the sequence of a single response where the stimulus interruption occurs (99). An intermittent stimulus may be seen as “continuous” if the recurrence frequency exceeds a certain threshold, whereas application of slower cycles below such threshold may elicit discordant recruitment of the compensatory responses. Further delineation of such complex responses to IH may permit the formulation of interventional strategies aiming at reducing the overall vulnerability of the young infant and child to apnea and sudden death (3).

Finally, it is also important to emphasize that clinical research involving young children and infants should be considered separately from that of adults and require the highest ethical standard for the researchers, because the pediatric subjects are often lack of complete understanding and cannot give informed consent to any procedure. Such ethical issues should be a primary concern for those who conduct IH research with infants and young children (100). The pediatric patients’ safety and welfare should always be the top priority of our research involving hypoxic stimulus. A proper choice of IHT regimens for children should take into account uneven and heterochronical development of different functions, in order to avoid the previously described negative effects of IH while maximizing its therapeutic potential.

## Conflict of Interest Statement

The authors declare that the research was conducted in the absence of any commercial or financial relationships that could be construed as a potential conflict of interest. The Review Editor Chin Moi Chow declares that, despite ongoing collaborations with the author Lei Xi, the review process was handled objectively.
